# Altered Structural and Functional Connectivity in Late Preterm Preadolescence: An Anatomic Seed-Based Study of Resting State Networks Related to the Posteromedial and Lateral Parietal Cortex

**DOI:** 10.1371/journal.pone.0130686

**Published:** 2015-06-22

**Authors:** Andrew J. Degnan, Jessica L. Wisnowski, SoYoung Choi, Rafael Ceschin, Chitresh Bhushan, Richard M. Leahy, Patricia Corby, Vincent J. Schmithorst, Ashok Panigrahy

**Affiliations:** 1 Department of Pediatric Radiology, Children's Hospital of Pittsburgh of UPMC, 4401 Penn Avenue, Floor 2, Pittsburgh, PA, 15224, United States of America; 2 Department of Radiology, University of Pittsburgh Medical Center (UPMC), 3950 Presby South Tower, 200 Lothrop Street, Pittsburgh, PA 15213, United States of America; 3 Brain and Creativity Institute, University of Southern California, 3620A McClintock Avenue, Los Angeles, CA 90089, United States of America; 4 Signal and Image Processing Institute, University of Southern California, Los Angeles, CA 90089, United States of America; 5 Department of Radiology, Children’s Hospital Los Angeles, Los Angeles, CA 90027, United States of America; 6 Department of Biomedical Informatics, University of Pittsburgh, Pittsburgh, PA, United States of America; 7 Twins Institute for Genetics Research, Montes Claros, Minas Gerais 39400–115, Brazil; 8 New York University Bluestone Center for Clinical Research, 421 1st Ave, New York, NY 10010, United States of America; The First affiliated Hospital of Xi’an Jiaotong University, CHINA

## Abstract

**Objective:**

Late preterm birth confers increased risk of developmental delay, academic difficulties and social deficits. The late third trimester may represent a critical period of development of neural networks including the default mode network (DMN), which is essential to normal cognition. Our objective is to identify functional and structural connectivity differences in the posteromedial cortex related to late preterm birth.

**Methods:**

Thirty-eight preadolescents (ages 9–13; 19 born in the late preterm period (≥32 weeks gestational age) and 19 at term) without access to advanced neonatal care were recruited from a low socioeconomic status community in Brazil. Participants underwent neurocognitive testing, 3-dimensional T1-weighted imaging, diffusion-weighted imaging and resting state functional MRI (RS-fMRI). Seed-based probabilistic diffusion tractography and RS-fMRI analyses were performed using unilateral seeds within the posterior DMN (posterior cingulate cortex, precuneus) and lateral parietal DMN (superior marginal and angular gyri).

**Results:**

Late preterm children demonstrated increased functional connectivity within the posterior default mode networks and increased anti-correlation with the central-executive network when seeded from the posteromedial cortex (PMC). Key differences were demonstrated between PMC components with increased anti-correlation with the salience network seen only with posterior cingulate cortex seeding but not with precuneus seeding. Probabilistic tractography showed increased streamlines within the right inferior longitudinal fasciculus and inferior fronto-occipital fasciculus within late preterm children while decreased intrahemispheric streamlines were also observed. No significant differences in neurocognitive testing were demonstrated between groups.

**Conclusion:**

Late preterm preadolescence is associated with altered functional connectivity from the PMC and lateral parietal cortex to known distributed functional cortical networks despite no significant executive neurocognitive differences. Selective increased structural connectivity was observed in the setting of decreased posterior interhemispheric connections. Future work is needed to determine if these findings represent a compensatory adaptation employing alternate neural circuitry or could reflect subtle pathology resulting in emotional processing deficits not seen with neurocognitive testing.

## Introduction

### Clinical Importance of Prematurity on Brain Development

Advances in perinatal care over the past few decades have increased the number of surviving preterm neonates. The effects of increased risk for medical conditions, learning disabilities, and psychological problems among early preterm infants lasting into adulthood have a visible impact on developed societies [1, 2]. Preterm birth may be associated with serious neurodevelopmental impairments related to deficits in executive function and attention [3–6]. Early prematurity significantly confers greater risk of autism spectrum disorders, and it is possible that these effects may also be observed in later preterm births [7]. While late preterm birth (generally between 34 and 36 6/7 weeks’ gestation) had traditionally been regarded as a relatively benign process due to a relatively lower neonatal morbidity and mortality compared to early preterm birth, the substantial developmental risks of late preterm birth, including cerebral palsy, are now better appreciated [8–10]. Developmental delay and increased academic difficulties are more common amongst even healthy children born late preterm with a risk of developmental delay approximately one-third higher than term infants [11, 12].

This study aims to ascertain possible functional connectivity and anatomical connectivity alterations in children born at late preterm that could relate to neurocognitive differences.

### Role of Default Mode Network in Brain Development

The default mode network (DMN) is a group of neuroanatomical structures acting in synchrony at-rest as indicated by resting state functional MRI (RS-fMRI) that are thought to integrate associative information and play a role in self-referential (introspective) thought activities [13, 14]. The late third trimester (32–40 weeks’ gestational age) serves as a critical period to form the foundation of the DMN marked by substantial increases in white matter architecture with gains from a rich-club network of highly clustered cortical hubs into a more globally integrated network [15, 16]. Importantly, these early DMN elements in infancy are mostly comprised of posterior structures such as posterior cingulate cortex (PCC), precuneus and parietal cortex with frontal structures emerging and increasing in connection strength alongside increased myelination across development [17–21]. Rudimentary posterior DMN connectivity patterns are observed in term infants, but not preterm infants (even at term-equivalent age), suggesting the late third trimester constitutes an important developmental epoch for DMN formation [22]. In addition, others have shown progressive formation of DMN connections between 29 and 40 weeks gestational age [23]. This finding is in distinction to the earlier emergence of other functional networks (e.g. auditory, visual and somatosensory) [18]. Highly clustered networks constituting early forms of resting state networks including salience, central-executive and default mode networks are apparent at 30 weeks’ gestational age, and these highly organized rich-club networks may be more vulnerable to injury during the late preterm period [16]. Therefore, targeted examination of resting state network formation during the late preterm period in this study is logical due to its later formation and concomitant vulnerability to insults related to prematurity.

### Vulnerability of Posterior Default Mode Network in Prematurity

Preterm birth-related effects demonstrate a posterior predilection with greater grey matter reductions in the parieto-occipital cortices and white matter injury predominantly affecting the posterior limb of the internal capsule and posterior portions of the corpus callosum [24–26]. Animal models of hypoxic-ischemic injury to model prematurity support this posterior injury bias with reactive astrocytes and microglia in these regions [27]. Because of the aforementioned predilection for preterm brain injury to the posterior portions of the cerebrum and the importance of the posterior DMN formation during infancy, this study focused on examining differences between children born at late preterm versus term within elements of the posterior DMN. To our knowledge, this is the first time that structural and functional connectivity have been examined simultaneously to assess the consequence of vulnerabilities during the late preterm period of brain development. We specifically focus on posterior cortical vulnerability in relation to resting state networks from a seed-based approach examining the posteromedial cortex and lateral parietal cortex.

## Materials and Methods

### Subjects

As part of a longitudinal international collaborative investigation of the genetic and environmental influences of prematurity on long-term neurocognitive functioning and health outcomes, 19 preadolescent pairs of twins (38 participants aged 9–13 years) were recruited from Minas Gerais, a developing region in Northeastern Brazil. The preadolescent twin pairs were recruited through an existing twin registry by newspaper advertisements. The registry is comprised of twins who had participated in prior studies investigating genetic influences on oral health [28, 29]. Sampling for this longitudinal program aims to balance zygosity (i.e., equal numbers of monozygotic and dizygotic twin pairs), prematurity, term birth, and gender.

### Ethics Statement

Research protocols were approved by local Institutional Review Boards in the United States (New York University, University of Pittsburgh and University of Southern California) and Montes Claros, Brazil (Universidade Estadual de Montes Claros). Written informed consent was obtained from study participants’ parents or legal guardians.

### Neurocognitive Measurements

Neurocognitive measures employed in this study included the Rey-Osterrieth Complex Figure Test (CFT), the Trail-Making Test (Children’s Version, Parts A and B), and selected non-verbal subtests from the NEPSY-II, including Memory for Designs (Immediate and Delayed), Affect Recognition, 3D Block Construction, Geometric Puzzles and Route Finding. Neurocognitive test administration was carried out in Portuguese by a Brazilian neuropsychologist after all test instructions had been translated into Portuguese. Moreover, an experienced, American neuropsychologist (JLW) was present for much of the neurocognitive test administration to ensure consistency and adherence to testing protocols. The neurocognitive tests were scored in accordance with published procedures [30] and US norms were used as a reference point for the NEPSY-II subtests. While abnormal results were not defined in reference to a normative sample, the use of US norms is not expected to affect the group level comparisons given that identical procedures were applied to both groups.

### Diffusion Tensor Imaging

#### Data Acquisition Protocol

Diffusion scans were acquired at 1.5 T with the following parameters: Spin-Echo EPI, TR = 6000 ms, TE = 90 ms, slice thickness = 2 mm, matrix = 112 X 112, FOV = 22.4 cm X 22.4 cm, SENSE factor = 2, b value = 1000 s/mm^2^, one scan acquired without diffusion weighting (b = 0 s/mm^2^) and 32 scans acquired with diffusion gradient direction according to the Philips Achieva 32-direction sequence.

#### Probabilistic Tractography Analysis

**Fig 1:**
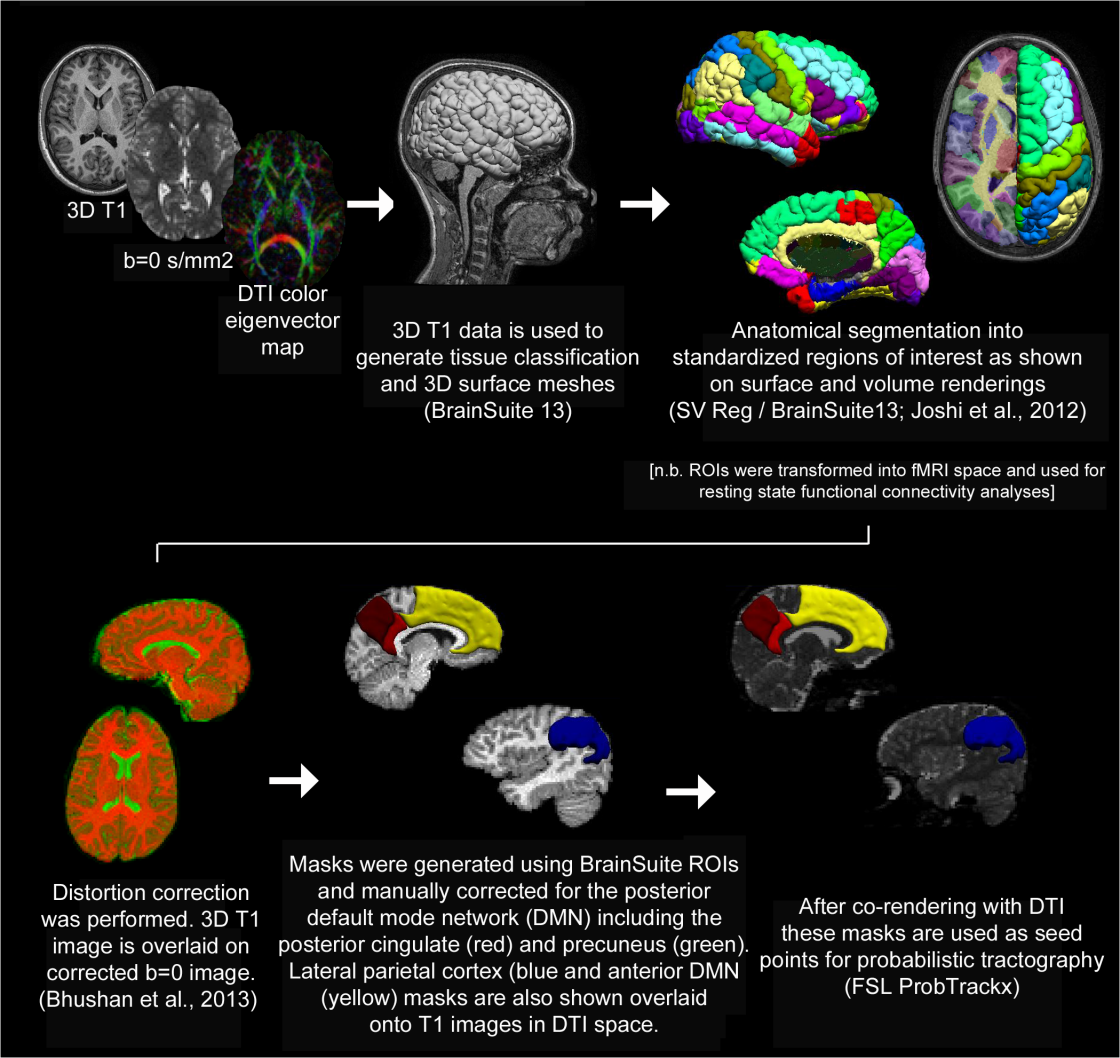
Summary of Tractography Methods. Probabilistic tractography comparison between groups was performed as summarized above [33, 87, 88].

Between-group probabilistic tractography analysis was carried out in a similar manner as a previously published method [31]. Diffusion tensors were estimated from diffusion scans after distortion correction and co-registration with segmented 3D-T1-weighted images using BrainSuite (http://brainsuite.usc.edu) including the SVReg atlas [32–34]. Seed regions were generated from anatomical labels for individual subjects and manually edited for each study to ensure precise correspondence with anatomical landmarks on a participant-specific basis. The posterior DMN seeds included the posterior cingulate and precuneus, both collectively and separately. Lateral inferior parietal cortex was also used as a seed related to the posterior DMN. These regions of interest were then applied as seed points for probabilistic tractography analysis using FSL ProbTrackx (FDT Toolbox, version 2.0; http://fsl.fmrib.ox.ac.uk/fsl/fsl-4.1.9/fdt/fdt_probtrackx.html). This process is depicted graphically in Fig 1. Following seed placement, streamlines representing tracts to and from these regions were reconstructed. 1000 streamlines were initiated from each seed voxel; the stopping criterion was a curvature threshold of 80 degrees. The number of streamlines through each voxel was divided by the total number of streamlines. These maps were spatially normalized into MNI space using routines in SPM8 (Wellcome Institute of Cognitive Neurology, London, UK). A white matter map was segmented from an anatomical T1-weighted image and then spatially normalized into MNI space using a pediatric white matter template; the same transformation was then used for the streamline maps. A GLM was used with preterm status the variable of interest; and sex and age as covariates of no interest. A clustered wild bootstrap (5000 repetitions, Rademacher distribution) was used [35] in order to appropriately handle the different sources of variance (e.g. within-family, between-family). For each bootstrap replication, the residual multipliers were kept the same for each voxel in order to accurately account for the effects of spatial autocorrelation. T-scores were determined as the mean over the bootstrap repetitions divided by the standard deviation. T-scores were then converted into Z-scores. A Monte-Carlo simulation [36] which estimates intrinsic spatial autocorrelation via “noise” maps obtained from the fit residuals was used to determine family-wise-error (FWE) statistical significance. FWE corrected p < 0.05 was determined to be at Z > 2.75, spatial filtering at σ = 3 mm, spatial extent threshold at 200 voxels (= 1600 mm^3^).

### Resting State Functional Connectivity

#### Data Acquisition Protocol

Resting-state BOLD was acquired on a Philips 1.5T Achieva scanner. Scan parameters were: TR = 3000 ms, TE = 50 ms, FOV = 21.1 cm X 21.1 cm, imaging matrix = 80 X 80, slice thickness = 4 mm, 30 slices acquired covering the whole brain, SENSE factor = 2, 100 volumes acquired for a total scan time of 5 minutes per run. Two scan runs were acquired: one with the child asked to keep his eyes open, and one with eyes closed. There was a brief time gap (approximately 15 seconds) between runs to allow the examiner to provide instructions to study participants to close their eyes.

#### Functional Connectivity Analysis

The data processing procedure closely followed that of Power et al. [37], shown to greatly minimize the risk of spurious results due to participant motion. Slice timing correction was performed using sinc interpolation with elimination of the first two TRs. The best reference frame for motion correction was found via minimization of an intensity-based cost function. Parameters for motion correction (affine) were found using a pyramid iterative routine [38]. The reference frames were transformed to the EPI template (in MNI space) using routines in SPM8 and resampled to 3 mm isotropic spatial resolution. A study-specific template was constructed via averaging the normalized reference frames across all participants and (rigid-body) coregistering the averaged reference template to the EPI template. Each reference frame was then normalized to the study-specific template. Each frame from each dataset was motion corrected (using the parameters found from above) and transformed into MNI space (using the transform found above) in a single step and normalized to global mean = 1000. The BrainSuite parcellation was also normalized into MNI space using the same transform. The mean voxelwise standard deviation between frames (DV) and the framewise displacement (FD) parameters (from the motion correction) were computed. Acceptable frames were those with DV < 30 and FD < 0.2 mm. The entire dataset was discarded if there were less than 40 usable frames. For each seed region (precuneus, posterior cingulate, posteromedial cortex, and angular gyrus), the seed time course was extracted by averaging over all voxels within the BrainSuite parcellation. Nuisance regressors (consisting of the parameters from the motion correction, linear and quadratic drift, and global signal mean) were regressed out of the seed time course. White matter and CSF signals were not included in the nuisance regressors, as their impact on results from group analysis has been shown to be minimal [37]. The seed time course was band-pass filtered with the pass band between 0.009 and 0.08 Hz, using only the retained frames, as the salient information in RS-fMRI is contained within this frequency band [39–43]. The seed time course was regressed on all voxels in the brain, using the nuisance regressors described above as covariates of no interest. The T-scores were retained for second level analysis. For second-level analysis, each participant was only included if there were at least 40 retained frames from each dataset (eyes open and eyes closed) and at least 100 total frames retained (corresponding to 5 minutes or more acquisition time). This resulted in retaining data from 25 (11 full-term, 14 preterm) out of the 38 participants while discarding data from the other 13 of the participants. A GLM was used with preterm status the variable of interest; and sex, age, eyes open status, square root of number of frames from the eyes open run, and square root of number of frames from the eyes closed run as covariates of no interest. A clustered wild bootstrap (1000 repetitions, Rademacher distribution) was used in order to appropriately handle the different sources of variance (e.g., within-participant, within-family, and between-family). For each bootstrap replication, the residual multipliers were kept the same for each voxel in order to accurately account for the effects of spatial autocorrelation. T-scores were determined as the mean over the bootstrap repetitions divided by the standard deviation. T-scores were then converted into Z-scores. A Monte-Carlo simulation [36], which estimates intrinsic spatial autocorrelation via “noise” maps obtained from the fit residuals, was used to determine family-wise-error (FWE) statistical significance. FWE corrected p < 0.05 was determined to be at Z > 3.25, spatial filtering at σ = 4 mm, spatial extent threshold at 100 voxels (= 2700 mm^3^).

## Results

### Patient Characteristics

We report results from a community-based cohort of low-to-moderate socioeconomic status preadolescent (ages 9–13) twin pairs born late preterm or at full term. This study is part of an international collaborative longitudinal research program to investigate genetic and environmental influences on prematurity, long-term neurocognitive functioning and general health outcomes. The preadolescents in this study were recruited from a city in Northeast Brazil (Montes Claros, pop. ca. 410,000). This region not only has a high incidence of natural multiple gestation pregnancies but also a high rate of late preterm birth coupled with limited healthcare resources [29]. As a result, none of the preadolescents had ever been hospitalized as infants in a neonatal intensive care unit. Therefore, this cohort provides a unique opportunity to analyze the longitudinal impact of prematurity-related stress during the late third trimester of brain development on the organization of developing neural systems. Late preterm birth was defined as gestational age between 34 and 36 weeks. The term controls group was defined as gestational age greater than 37 weeks without perinatal complications. There were a total of 38 pre-adolescent children recruited with a median age of approximately 10 years for late preterms and approximately 10 years for term controls. Thirteen resting-stated BOLD MRI studies were removed for technical reasons (motion-related artifacts) resulting in a total of 25 subjects for final functional connectivity analysis (11 preterm and 14 full-term preadolescent children).

### Neurocognitive Assessment

The neuropsychological test battery performed by all participants included the Rey-Osterrieth Complex Figure Test (CFT), the Trail Making Test (children’s version), and selected subtests of the NEPSY-II assessing visuospatial, visuo-perceptual, and memory functions: Affect Recognition, Memory for Designs, Block Construction, Geometric Puzzles, and Route Finding. No significant difference in cognitive performance was seen between preterm and term preadolescents after controlling for age (MANCOVA, F (12,27) = 0.735, p > 0.5). A nominally significant difference in the direction of the preterm preadolescents outperforming the term preadolescents was detected only on Block Construction (F (1,37) = 4.131, p = 0.049), not surviving correction for multiple comparisons.

### Resting State Functional Connectivity Results

#### Posteromedial Cortex

**Fig 2:**
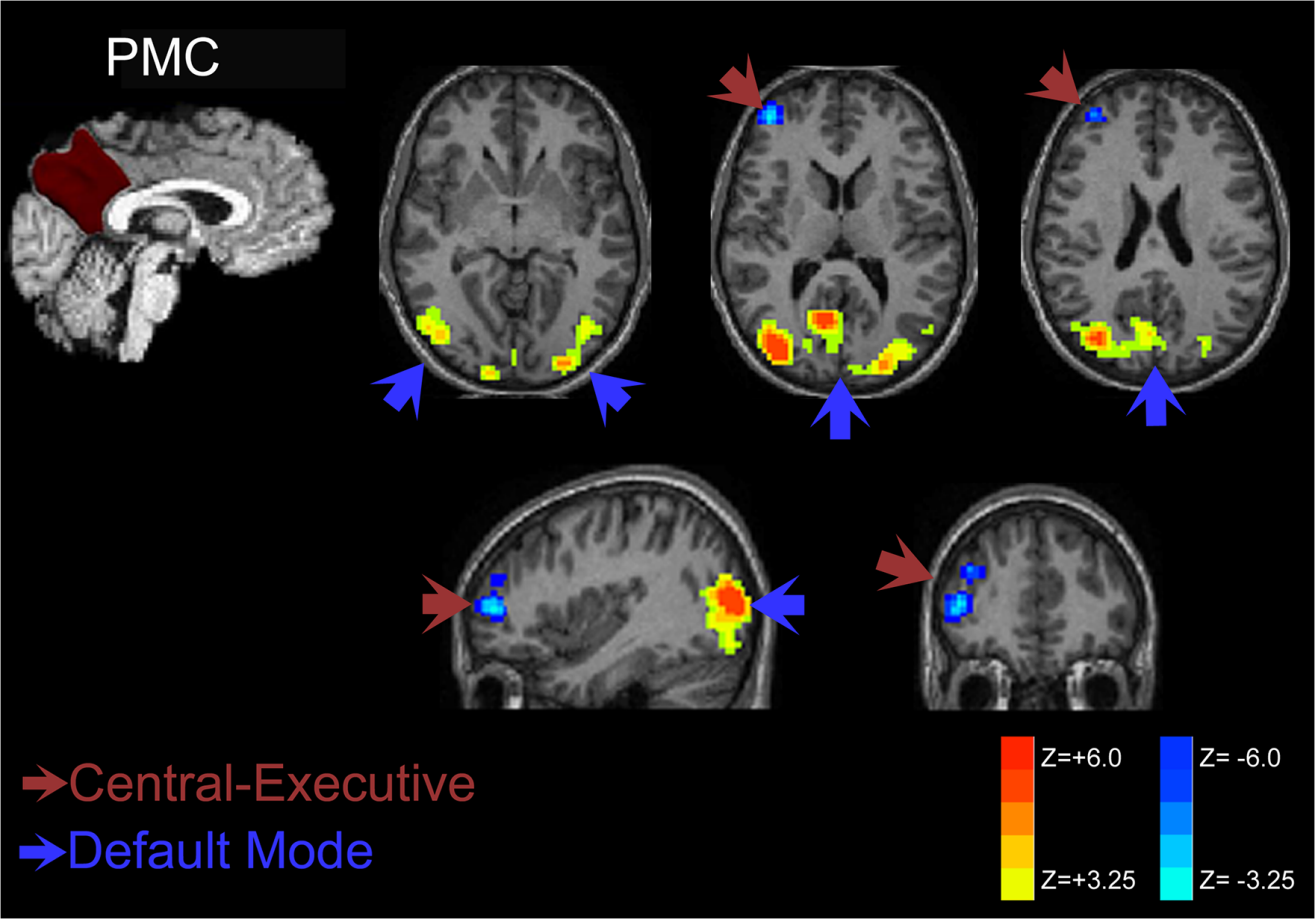
Posteromedial Cortex Connectivity Comparison of Late Preterms versus Term Control Children. Representative plots on standard anatomic images demonstrate increases in functional connectivity in late preterms within the posterior DMN components. There is significant decreased connectivity to components of the executive network (right frontal). These findings are consistent with late prematurity-related posterior DMN hyperconnectivity and increased anti-correlation (decreased functional connectivity) to the central-executive network.

Late preterm individuals during preadolescent childhood demonstrate increased functional connectivity when seeding from the PMC as shown in Fig 2. Increased functional connectivity strength was noted within the posterior DMN of the parietal and occipital cortices. Significantly increased anti-correlation was present within the dorsolateral prefrontal cortex for late preterms – a key component of the central-executive network.

**Fig 3:**
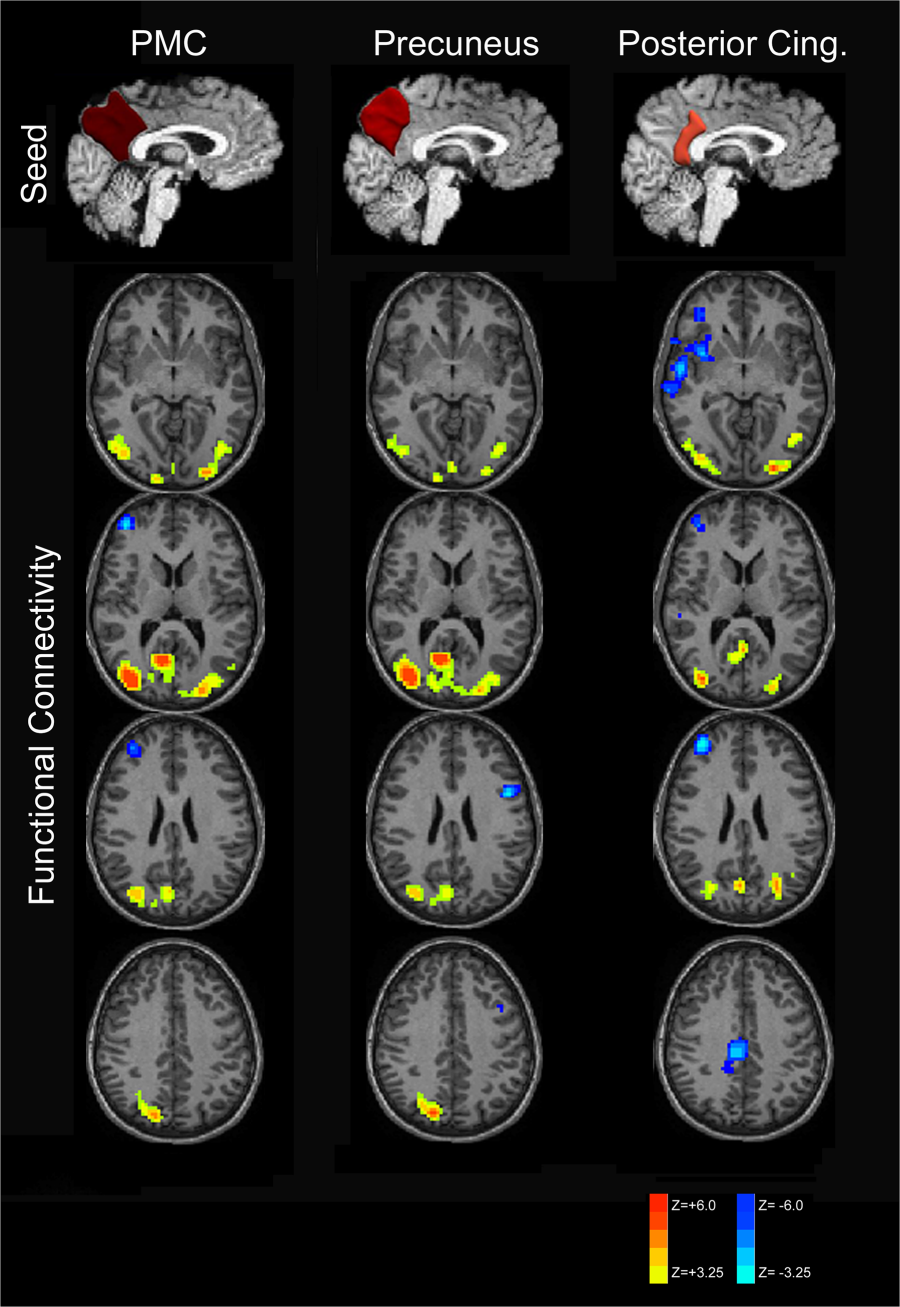
Subdivision of PMC functional connectivity into precuneus and posterior cingulate components. RS-fMRI analysis of the PMC demonstrates increased connectivity in the posterior default mode network for PMC and its components. Anti-correlation with the salience and central-executive networks was only observed with the posterior cingulate seed and not when seeding the precuneus, however.

As shown in Fig 3, the patterns of connectivity increases in late preterm individuals demonstrate similar increased connectivity within the posterior parietal and occipital cortices of the posterior DMN for both precuneus and posterior cingulate seeding. Posterior cingulate seeding demonstrated slightly greater increased connectivity within the posterior DMN. More importantly, the posterior cingulate cortex seeds demonstrated anti-correlation with the salience network components (insular cortex) and central executive network (prefrontal cortex) not observed with precuneus seeding. This finding suggests decreased connectivity involving both the salience and central-executive networks in late preterm children.

**Fig 4:**
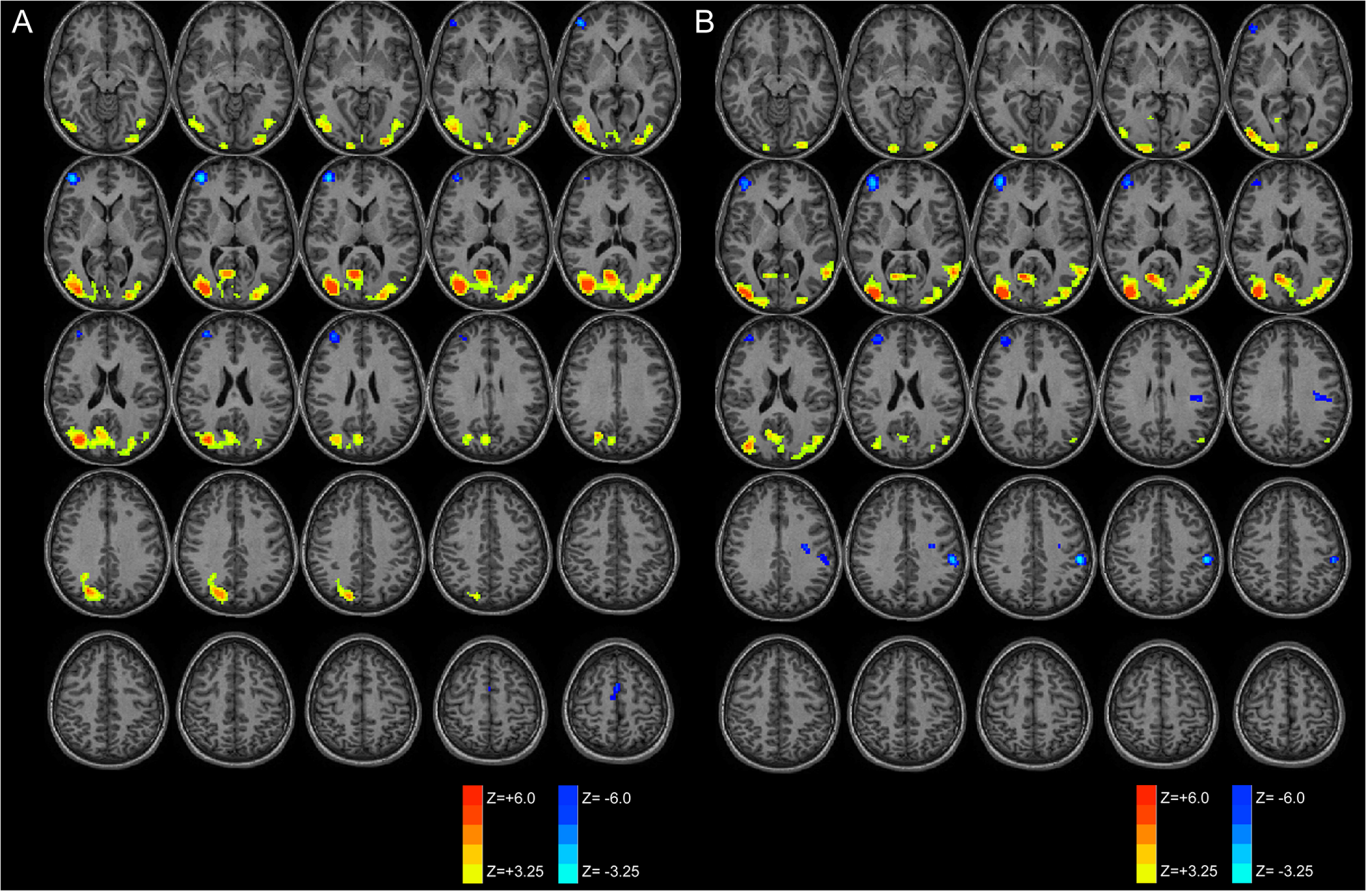
Laterality of functional connectivity patterns of the posteromedial cortex. Increased functional connectivity is demonstrated for both left (A) and right (B) PMC seeds within the posterior DMN with greater connectivity for the right hemispheric seeds. Decreased connectivity (increased anti-correlation) is seen within the central executive network.

Strength of increased functional connectivity within the posterior DMN was greater for the right hemispheric seeds compared to left-sided seeding as displayed in Fig 4 and verified by statistical testing (data not presented).

#### Lateral Parietal Cortex

**Fig 5:**
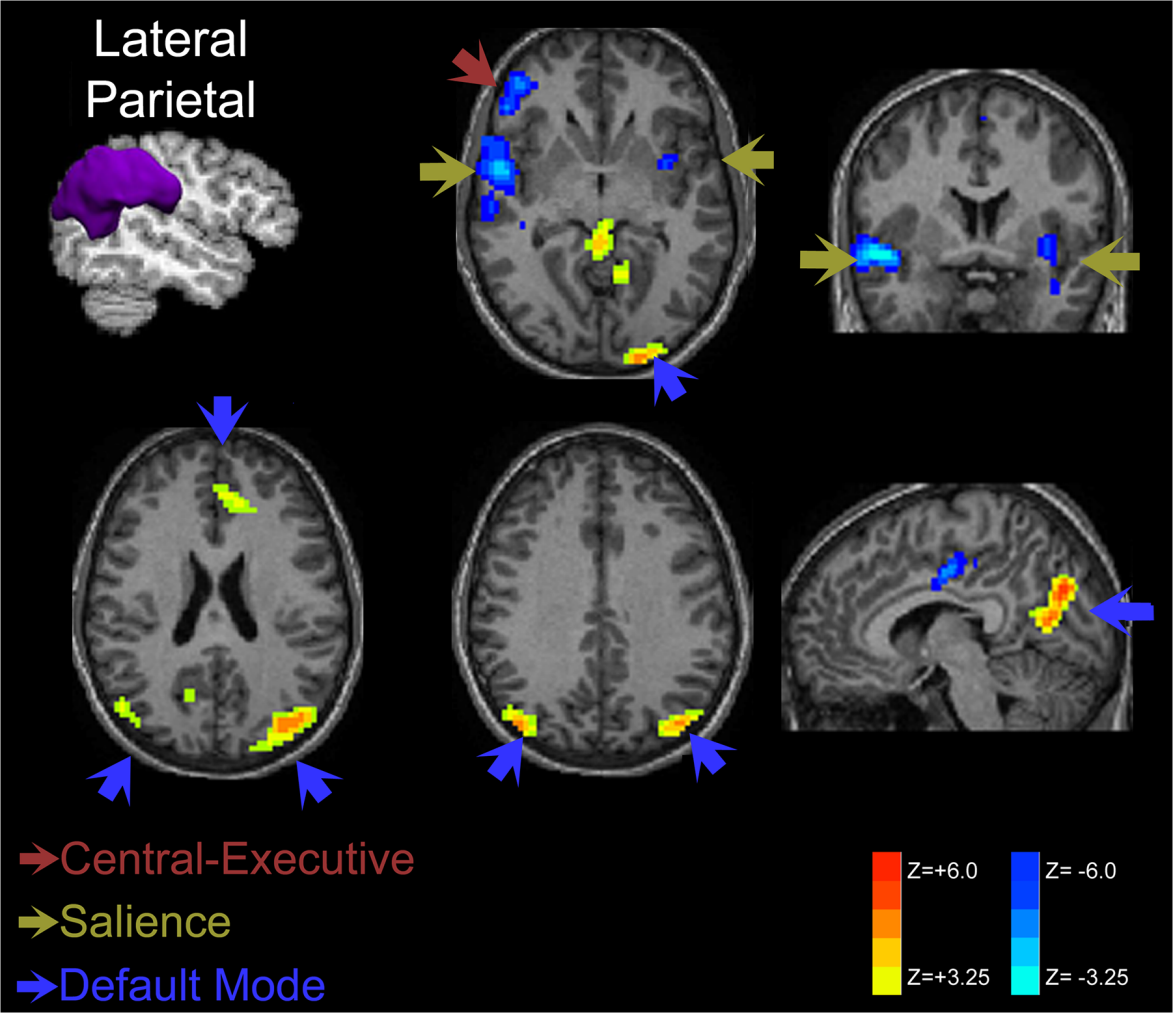
Lateral Parietal Cortex Connectivity Comparison of Late Preterms versus Term Control Children. Increased connectivity within late preterm children is present predominantly within the posterior DMN when seeded from the angular and superior marginal gyri, although some increased connectivity was observed within the anterior cingulate cortex of the DMN. Decreased connectivity was observed within components of the salience network (insular cortex) and central executive network (prefrontal).

Hyperconnectivity of the DMN in late preterm children was also demonstrated when seeding from the lateral parietal cortex (superior marginal and angular gyri) as shown in Fig 5 with increased connectivity of posterior DMN structures as well as the anterior cingulate cortex. Components of the right salience network (insular cortex) and executive network (frontal cortex) demonstrate increased anti-correlation when seeded from the parietal cortex similar to the posterior cingulate cortex seed results. Increased connectivity to predominantly posterior DMN structures from lateral parietal seeding was present for both sides, but overall magnitude was greater for the left seed.

### Probabilistic Tractography Results

#### Posteromedial Cortex

**Fig 6:**
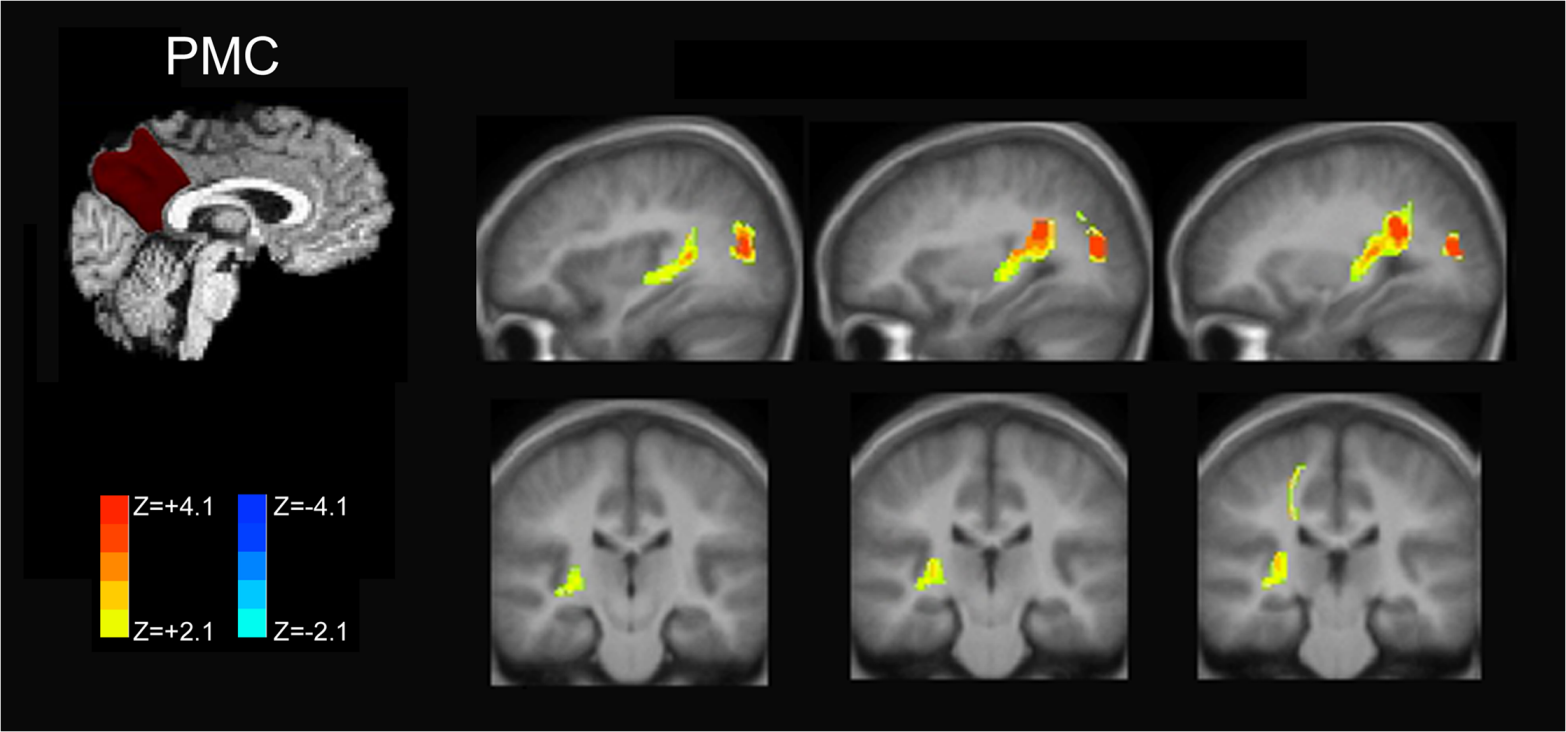
Increased Structural Connectivity related to the Posteromedial Cortex within IFOF and ILF. Using probabilistic tractography from the right PMC seed, increased streamlines were demonstrated within the right inferior longitudinal (occipitotemporal) fasciculus (ILF) and overlapping inferior fronto-occipital fasciculus (IFOF). These increased streamlines overlapped with the axon-rich sagittal stratum. Color bars indicate increased streamlines (+2.1 to +4.1) and decreased streamlines (-2.1 to -4.1).

Increased streamlines were demonstrated within the right inferior longitudinal (occipitotemporal) fasciculus (ILF) and overlapping inferior fronto-occipital fasciculus (IFOF) as shown Fig 6. These areas of increased structural connectivity overlap with the sagittal stratum, which constitutes an axon-rich bundle to the occipital lobe including ILF, IFOF and posterior thalamic radiation (PTR) [44]. The ILF connects the occipital lobe with the anterior portions of the temporal lobe [45]. The ILF contains both direct fibers the occipital lobe to the temporal lobe as well as short association fibers (subcortical U fibers) as an indirect occipitotemporal projection system [46]. Decreased white matter integrity within the ILF is thought to generate difficulties with object recognition, cognition, visual memory, emotional processing and has been implicated in visual hallucinations in schizophrenia [45–49]. Overlapping with this fiber bundle is the inferior fronto-occipital fasciculus (IFOF, also referred to as the inferior occipitofrontal fasciculus), which connects the orbito-frontal, temporal and occipital regions. This is thought of as a pathway for integrating association cortices with the prefrontal cortex [50]. Both ILF and IFOF share projections from the posterior temporal and occipital lobes [50, 51].

**Fig 7:**
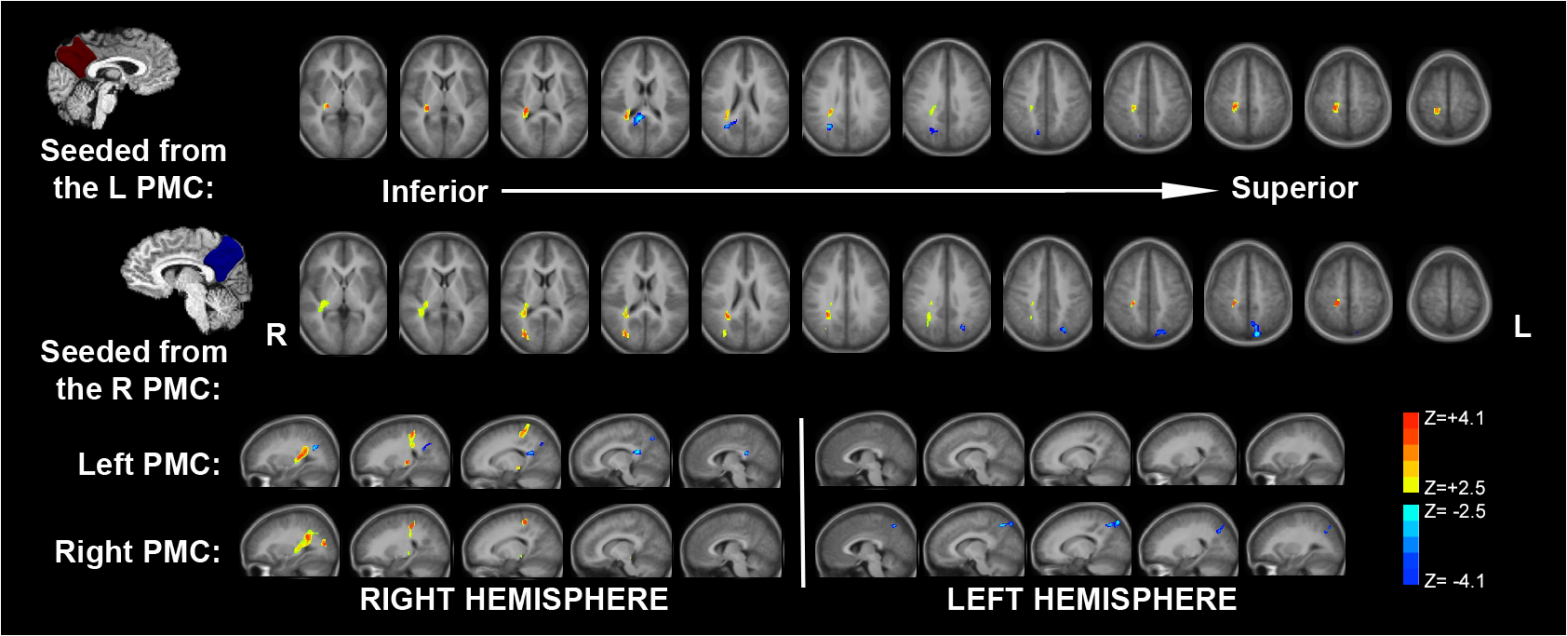
Comparison of Tractography Differences between the Right and Left Posteromedial Cortex. Seeding from both the right and left posteromedial cortex demonstrated increased streamlines within the right ILF/IFOF, but the magnitude of increased streamlines was greater for the right PMC seed. Decreases within the splenium (mostly within the more right aspect) were seen with left seeding while at the same time significant increases were seen within subcortical white matter near the right lateral parietal cortex. Right seeding also showed decreases within the left posterior parietal subcortical white matter. Color bars indicate increased streamlines (+2.1 to +4.1) and decreased streamlines (-2.1 to -4.1).

In seeding the right and left posteromedial cortex as depicted in Fig 7, both seeds resulted in significantly increased streamlines within the late preterm children compared to control children within the right ILF/IFOF as previously discussed although the left seed streamlines appear less substantial. Decreases were seen in the posterior corpus callosum (splenium) for the left PMC seed, implying decreased interhemispheric connectivity. Decreases for the right PMC seed are demonstrated within the left posterior parietal-occipital white matter. Left PMC seeding, on the other hand, demonstrated increased streamlines within subcortical white matter in the region of the right lateral parietal cortex, implying increased anatomical connectivity to the default mode network hub.

#### Lateral Parietal Cortex

**Fig 8:**
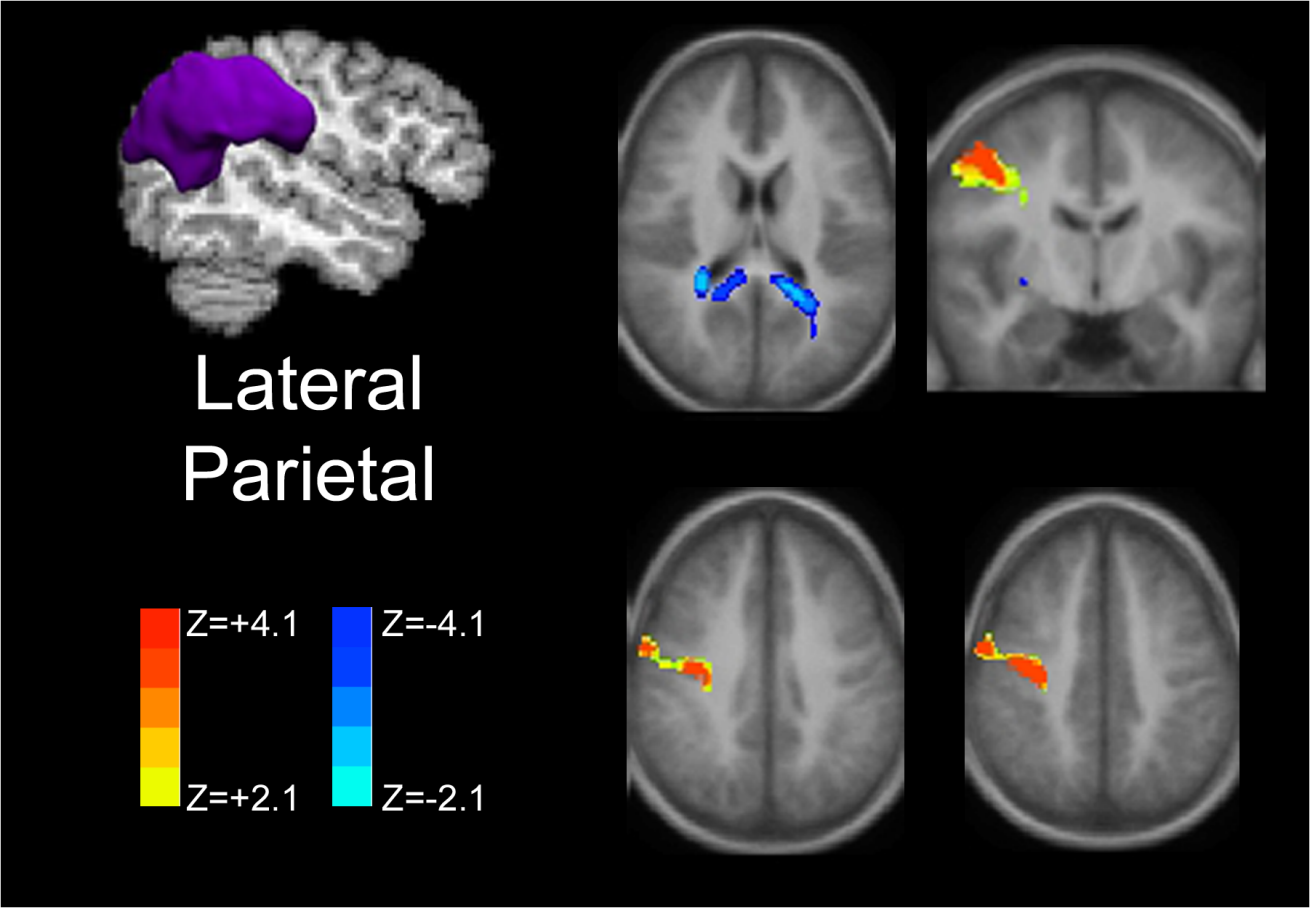
Lateral Parietal Cortex Structural Connectivity. Anatomic-based seeding from the lateral parietal cortex (superior marginal and angular gyri) demonstrates decreased streamlines involving the splenium of the corpus callosum and increased streamlines within the frontal white matter near the supplementary motor area. Color bars indicate increased streamlines (+2.1 to +4.1) and decreased streamlines (-2.1 to -4.1).

Seeding from the right lateral parietal cortex as shown in Fig 8 demonstrated reduced streamlines within the posterior corpus callosum as in the case of the left PMC seed, suggesting a common reduction in interhemispheric connectivity. Increased streamlines for late preterm children are noted within the subcortical white matter near the supplementary motor area.

## Discussion

### Functional Hyperconnectivity of the posterior DMN in Late Prematurity

Late preterm birth is increasingly recognized as an important influence in brain development with documented impairments in learning and neuropsychological performance [52]. In our examination of preadolescent children born late preterm imaging anatomical and functional connectivity highlights key differences even in the absence of differences in results on neuropsychological testing. Connectivity was increased in late preterm children predominantly within the posterior default mode network and associated with greater anti-correlation to right-sided components of the salience and executive networks. These findings are suggestive of key functional connectivity alterations in late prematurity without significant differences in cognitive functioning.

During the late third trimester, what mainly shapes brain development is the organization and reorganization of connections between existing neurons, as neurogenesis is considered largely complete [53]. At the macroscopic level, the developing connectivity is reflected in the dramatic changes in brain mass and volume, which increase by 35% and 50% respectively in the last six weeks of fetal development [54]. At a microscopic level, the developing connectivity is reflected in the outgrowth of axons and the formation of synapses between newly formed axons and dendrites—first, constructing thalamocortical networks and later, corticocortical networks, including the DMN [55]. In contrast to the early preterm period (i.e., < 32 weeks), which is characterized by an ingrowth of thalamocortical connections into the developing cortex, the late preterm period is characterized by an ingrowth of callosal and long-range intrahemispheric corticocortical afferents into the developing cortex. Recent work has shown that signaling mechanisms that guide such axonal ingrowth as well as synaptogenesis are at low levels in late prematurity [56]. While several studies have shown how premature birth during the early preterm period (26–32 weeks) affects the development of visual, auditory, and somatosensory networks, it is less certain how the environmental stress of *late* premature birth (32–36 weeks) may affect the development of the DMN and how such possible alterations can affect brain organization.

Recent progress has been made in understanding the early formation of resting state networks during late gestation. Some research posits a rich-club network of highly clustered cortical hubs within the infant brain develops by 30 weeks' gestation [16]. The late gestational period may transition from a small number of densely connected hubs into a more globally integrate network [15]. Interestingly, these rich-club regions match those areas of hyperconnectivity represented in our study—the dorsal/medial frontal cortex, parietal cortex, precuneus, hippocampus, insula, cingulate cortex, angular and fusiform gyri [15]. During the final trimester, connections between these rich-club hubs and the remainder of the cortex increase [16]. Expected normal maturation should result in a broadly distributed network with decreased connectivity within clustered rich-club networks. Instead, we report increased connectivity from lateral parietal and PMC seeds to components of these rich-club networks, implying preserved connections to these less mature networks. Therefore, the observation of hyperconnectivity in rich-club networks during late preterms during childhood in our study may indicate a failure to progress beyond these initial rudimentary resting networks to a more mature, distributed network.

Despite lacking significant cognitive differences between preadolescent children born late preterm versus at term in this study, there remains valuable information regarding possible anatomical and functional connectivity explanations for increased risk of developmental delay and cognitive disability with prematurity. Because of an increased risk of autism with preterm birth, preterm birth could share changes in brain connectivity with autism [57, 58]. In addition, some investigators attribute autistic behavior to a lack of integrating associative information – a function of the DMN [59]. In autism, increased dendritic spine density has been observed within the frontal, temporal and parietal cortex that correlated inversely with cognitive function [60]. These finding suggests a possible pathophysiological link to decline in cognitive function in autism with greater dendritic spine density thought to correspond to excessive excitatory connectivity [61]. Functional connectivity studies are varied in autism, but some show a decrease in connectivity between the anterior cingulate with the PMC in autism; findings of a lack of anterior-posterior connectivity in the DMN may also be seen in preterm infants [62–64]. On the other hand, increases in task-related activation in autism have been observed within the parietal cortex [65].

Increased excitatory activity or, as suggested in our study, excess functional hyperconnectivity within posterior DMN structures may relate to disruption of normal synaptic pruning that is expected to occur postnatal development, but may be altered in a variety of conditions such as autism and obsessive-compulsive disorder [66, 67]. In our study, increased functional connectivity within the posterior DMN in late preterms was seen without significant abnormalities on neurocognitive testing. In an animal model of prenatal valproic acid exposure, neocortical pyramidal neurons were found to have increased local connectivity that was more sensitive to excitation and more active [68]. Therefore, the absence of normal synaptic pruning may lead to neural hyperactivity. Work examining young rhesus monkeys previously demonstrated effective postnatal pruning with a substantial decrease in axonal connections from birth. Prematurity could effectively halt this selective axonal pruning and thereby explain findings of hyperconnectivity that persist into childhood. [69, 70]. More recent work in a murine model shows that deficits in synaptic pruning by microglia lead to immature brain circuitry [71].

While the clinical significance of hyperconnectivity observed in this study is not definitively associated with cognitive dysfunction, the context of these findings combined with that of others in autism and prenatal injury strongly suggests that a developmental deficit in pruning of local connections may be responsible for neurocognitive deficits seen in late prematurity. This lack of pruning may occur with greater preponderance in the posterior regions as indicated by predominant hyperconnectivity within the posterior default mode network and established posterior cortical vulnerability.

### Selective Increased Anatomical Connectivity in Late Prematurity

Probabilistic tractography differences were less pronounced in comparison to the previously described functional connectivity findings as expected in this group of late preterms without significant cognitive deficits. Nevertheless, in this study, we observe selectively increased streamlines in our probabilistic tractography analysis of later preterm versus term infants within the region of the right inferior occipito-frontal fasciculus (IFOF) and right inferior longitudinal (occipito-temporal) fasciculus (ILF). In addition, the left PMC showed increased streamlines within right parietal white matter. These increases occurred in the setting of decreased streamlines within the splenium of the corpus callosum from the left PMC and right lateral parietal cortex, thereby implying deceased interhemispheric connections.

Previously, others [72] have noted increased anisotropy in the sagittal strattum (which includes IFOF and is adjacent to the ILF) in preterms. These changes were bilateral but more substantial within the right, interestingly. More recent work by Feldman and colleagues shows increased fractional anisotropy (FA) within the right inferior fronto-occipital/inferior longitudinal fasciculi posteriorly and bilateral superior longitudinal fasciculi in high functioning preterms during adolescence [73]. Others previously showed increased FA within the inferior fronto-occipital fasciculus and uncinate fasciculi in very preterm individuals at young adulthood [74].

On the other hand, earlier voxel-based morphometry studies of earlier preterms show decreased white matter volume in the longitudinal fasciculi both during infancy and early adolescence [75, 76]. In preterm VLBW infants with deficits, FA is lower in the inferior fasciculus and inferior fronto-orbital fasciculus [77], but this is not a comparable study to our group of late preterms and in Skranes’ group, most patients had ventriculomegaly and other abnormalities. Moreover, it is uncertain whether there is initial insult in this region indicated by low FA that subsequently recovers during development with later increases when these preterm infants are imaged during later childhood or early adulthood.

We propose that the finding of increased right IFOF/ILF streamlines suggests compensatory increased anatomical connectivity within the right hemisphere. The conventional pathway for semantic language processing follows the inferior fronto-occipital fasciculus (IFOF) with the inferior longitudinal fasciculus (ILF) constituting an indirect pathway [78]. Prior work in preterms during adolescence hypothesizes that differences represent recruitment of an alternative language circuit with bilateral ventral pathway involvement [79, 80]. These findings of bilateral language pathway formation with prematurity can be thought of as an arrest of normal left-side language dominance occurring during the final trimester of prenatal development [81]. In a study of children with stuttering, only the right inferior longitudinal fasciculus and uncinate fasciculus demonstrated increased anatomical connectivity with higher FA in stuttering children [82]. In general, increased FA within the uncinate and arcuate fasciculi corresponds to better performance on language tasks [83]. Therefore, increased streamlines in our probabilistic tractography in this region in late preterms with normal cognitive performance may suggest a pattern of compensatory neural circuitry that is consistent with others' work [73].

Another possible explanation for selective increased streamlines within the IFOF and ILF in late preterm infants may represent aberrant white matter development that affects brain function not assessed by conventional neuropsychological tests. Prior work in William’s syndrome has shown similar increased FA within the IFOF that is thought related to increased myelination [84]. While these patients had changes within the right fusiform gyrus that corresponded to social cognition scores, no significant correlations were observed for white matter changes including the IFOF and test results [84]. Moreover, ex-preterms have been found to have social deficits that related to alterations within emotional processing networks [85]. Hyperconnectivity is also documented in autism and is directly correlated with social dysfunction [86]. Our work adds to increasing evidence suggesting hyperconnectivity within emotional processing networks may serves as the basis for social deficits in ex-preterm individuals.

### Study Limitations

This study included individuals who were born at late preterm versus term gestational age and examined brain development during the preadolescent period. One important aspect of this study population is the lack of early perinatal intervention in a preterm population from a poor, developing region [28, 29]. While this allows us to observe the effects of late prematurity without bias from early intervention, our observations of differences may not correlate with those of late preterm infants from higher socioeconomic regions, who would be expected to exhibit less significant differences in functional and structural connectivity. In addition, other unknown environmental variables could play a role in differences between groups. Nevertheless, as all study participants were recruited from the same impoverished region of a developing country, differences between the groups would be expected to reflect the effects of late preterm birth without early intervention although socioeconomic status was not formally measured.

In analyzing this data *post hoc*, there was a slight but statistically significant increase in the number of included scans in the eyes-open condition for the preterm group, although not for the eyes-closed condition. Accordingly, there was a slight but significant difference in the framewise displacement (FD) parameter between groups in the eyes-open condition (mean FD = 0.10 for preterms, mean FD = 0.11 for terms) though not for the eyes-closed condition (mean FD = 0.093 for preterms, mean FD = 0.097 for terms). As part of the functional connectivity data processing, WM and CSF values were not included as regressors in the analysis as they were not shown to significantly change the results. These minor technical limitations are not expected to significantly affect the results of this study due to the rigorous frame inclusion criteria, however future studies may benefit from these additional data analysis strategies.

Serial data examining patterns of development would strengthen our understanding developmental phenomena related to late preterm birth by looking at dynamic changes over time within the individual rather than at one point in time. Despite promising results from an increasing body of literature, both methods are indirect indicators of functional and anatomical connectivity, respectively. Moreover, conventional DTI is limited by its inability to distinguish tracts within axon-rich crossing-fiber regions, a limitation that may be overcome with future work employing diffusion spectrum imaging.

One other key aspect of this study was the use of participant-specific anatomic parcellation to accurately identify the function of the anatomic PMC structures. Function-based parcellation may yield differing results that could add further detail to our study. In addition, the use of other methods such as graph theory or independent components analysis could be helpful to validate our findings. Moreover, future work comparing our findings with those of earlier preterm infants in a different setting would enrich our understanding of the magnitude of brain development alterations related to prematurity within the posterior DMN and other critical neuroanatomical regions.

## Conclusions

Our findings support appreciable abnormalities in brain development in infants born late preterm even in the absence of detectable differences in neuropsychological performance. Thus, the late prenatal period represents a key timeframe for the formation of mature resting state networks and disruption of these neural connections may incite increased local functional connectivity within the posterior DMN, greater anti-correlation with salience and executive networks and lead to the strengthening of additional axonal pathways within the inferior longitudinal fasciculus and inferior fronto-occipital fasciculus that serve as compensatory alterations or pathologic sequelae that could affect emotional processing and social function not adequately assessed on routine neuropsychological testing.
